# Quantifying cancer- and drug-induced changes in Shannon information capacity of RTK signaling

**DOI:** 10.1038/s41598-025-23075-y

**Published:** 2025-11-10

**Authors:** Paweł Nałęcz-Jawecki, Lee Roth, Frederic Grabowski, Sunnie Li, Marek Kochańczyk, Lukasz J. Bugaj, Tomasz Lipniacki

**Affiliations:** 1 Institute of Fundamental Technological Research, Polish Academy of Sciences, Warsaw, 02-106 Poland; 2 Department of Bioengineering, University of Pennsylvania, Philadelphia, PA 19104 USA; 3 Department of Statistics, Rice University, Houston, TX 77251 USA

**Keywords:** Channel capacity, MAPK pathway, Cancer, EML4-ALK oncogene, Optogenetics, Growth factor signalling, Information theory, Applied mathematics, Cancer

## Abstract

Cancer can result from abnormal regulation of cells by their environment, potentially because cancer cells may misperceive environmental cues. However, the magnitude to which the oncogenic state alters cellular information processing has not been quantified. Here, we apply pseudorandom pulsatile optogenetic stimulation, live-cell imaging, and information theory to compare the information capacity of receptor tyrosine kinase (RTK) signaling pathways in EML4-ALK-driven lung cancer (STE-1) and in non-transformed (BEAS-2B) cells. The average information rate through RTK/ERK signaling in STE-1 cells was less than 0.5 bit/hour, compared to 7 bit/hour in BEAS-2B cells, but increased to 3 bit/hour after oncogene inhibition. Information was transmitted by 50–70% of cells, whose channel capacity (maximum information rate) was estimated through in silico protocol optimization. In BEAS-2B cells, channel capacity of the parallel RTK/calcineurin pathway surpassed that of the RTK/ERK pathway. This study highlights information capacity as a sensitive metric for identifying disease-associated dysfunction and evaluating the effects of targeted interventions.

**Supplementary Information:**

The online version contains supplementary material available at 10.1038/s41598-025-23075-y.

## Introduction

 The existence of oncogenes throughout healthy human tissues underscores decades of studies showing that cancer arises not only from the presence of an oncogene but also from improper regulation of precancerous cells by their environment^1,2^. Such misregulation may result not only from changes in the cell’s environment but also from changes in how a cell responds to cues from that environment. However, how an oncogenic state alters a cell’s perception of its environment remains largely unexplored.

**Fig. 1:**
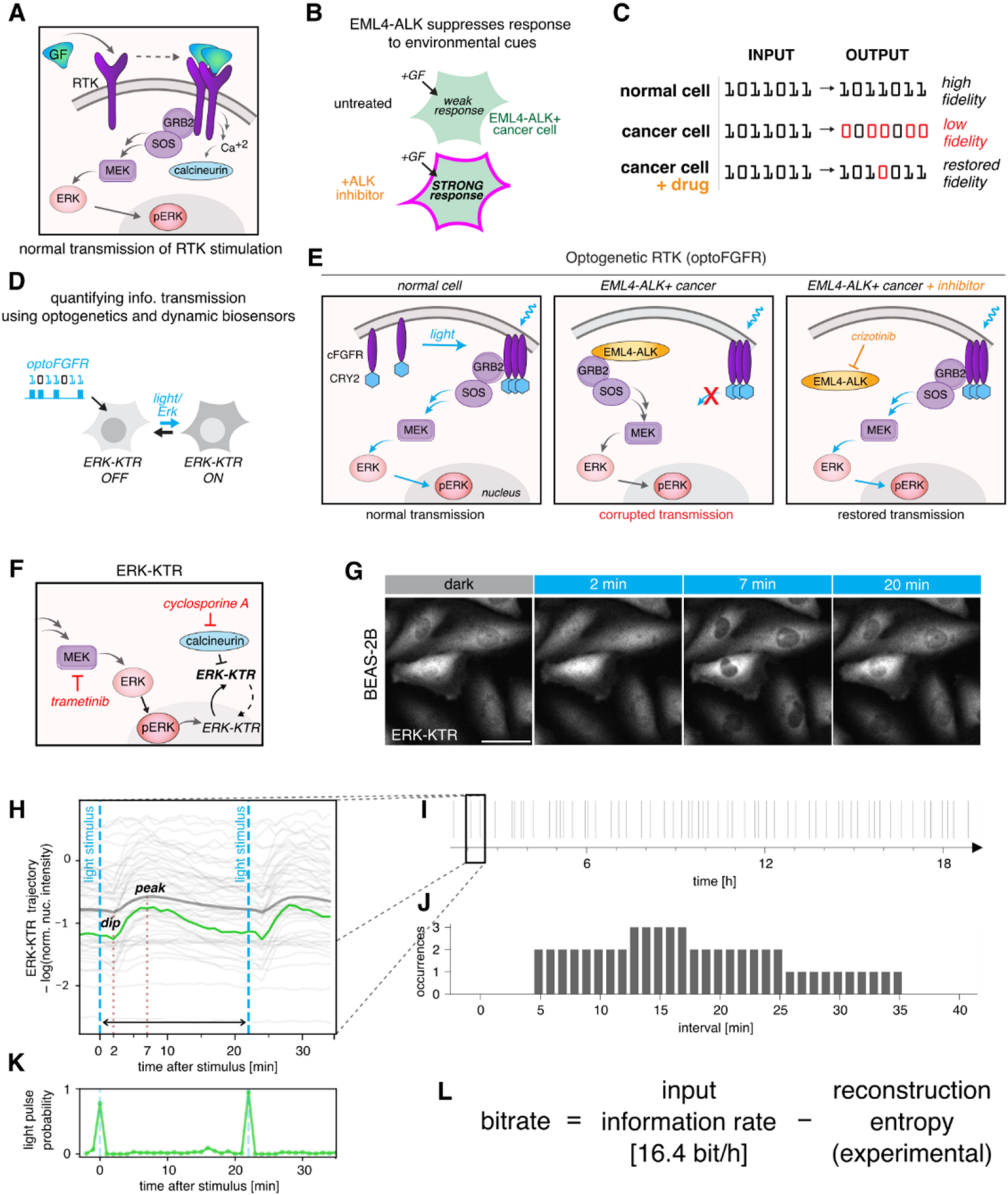
Experimental setup. (**A**) After binding to a growth factor (GF), receptor tyrosine kinases (RTKs) multimerize and signal to ERK via RAS, RAF, and MEK (the RTK/ERK pathway). Simultaneously, RTKs trigger the calcium-calcineurin pathway (RTK/calcineurin pathway). (**B**) The EML4-ALK oncogene suppresses a cell’s ability to respond to environmental cues such as growth factors. The ALK inhibitor (ALKi) restores responsiveness. (**C**) In normal cells, activity pulses are accurately transmitted by the RTK/ERK pathway from receptors to the nucleus. EML4-ALK-positive cancer cells transmit environmental information with low fidelity, which can (to some extent) be restored by ALKi. (**D**) Pulsatile optogenetic FGFR (optoFGFR) stimulation and fluorescent biosensors of signaling allow the quantification of information transmission through the RTK/ERK signaling axis. The ERK-KTR biosensor translocates between the nucleus and the cytoplasm upon ERK activation/inactivation. (**E**) OptoFGFR, in response to blue light, recapitulates endogenous RTK activation. Cell responsiveness to RTK signals may be suppressed by EML4-ALK, which associates with and sequesters GRB2-SOS. Signal transmission can be restored by ALKi, which liberates GRB2-SOS from EML4-ALK. (**F**) ERK-KTR migrates to the cytoplasm upon phosphorylation by ERK, and in the reverse direction after dephosphorylation by calcineurin. Inhibitors of calcineurin (cyclosporine A) or MEK (trametinib) were used to study the two pathways in isolation. (**G**) ERK-KTR cytoplasmic–nuclear shuttling after stimulation with a light pulse in BEAS-2B cells. Fluorescence was recorded with a one-minute resolution. Scale bar = 20 μm. (**H**) Light stimulus leads to ERK-KTR phosphorylation, resulting in cytoplasmic translocation (a peak is observed approximately 7 min after the light pulse), preceded by a calcineurin-mediated ‘dip’ observed 2 min after the light pulse in BEAS-2B cells. The thin gray lines represent single-cell preprocessed ERK-KTR trajectories, and the thick gray line represents their average. The green line shows a selected single-cell trajectory for which the (posterior) probability of a light pulse is given in panel K. See Methods for details of ERK-KTR trajectory preprocessing. (**I**) The cells were stimulated with a pseudorandom series of light pulses. Information is encoded in intervals between subsequent pulses. (**J**) The distribution of intervals between stimulation pulses was chosen on the basis of preliminary experiments to maximize the bitrate. (**K**) Single-cell ERK-KTR trajectories were used for probabilistic reconstruction of the input signal, i.e., to predict the probability of a light pulse occurrence at each time point throughout the experiment at a one-minute temporal resolution. (**L**) The numerically estimated entropy of the probabilistic reconstruction was used to compute the rate of information transmission through the pathway.

Biochemical signaling pathways regulate a cell’s response to its environment. For example, ligand stimulation of receptor tyrosine kinases (RTKs) triggers the RAS/RAF/MEK/ERK pathway (Fig. 1A), referred to here as the RTK/ERK pathway, which is responsible for cell proliferation, survival, and differentiation^3^. The RTK/ERK pathway is often stimulated in transient pulses and regulates processes that require high spatiotemporal resolution, such as collective cell migration and embryogenesis^4–6^. RTK signaling also activates additional pathways, including the PI3K/AKT/mTOR and calcium signaling pathways, both of which are also dynamic and important for cell growth and division^7–10^. In line with the central role of RTKs in cell proliferation, mutations in RTKs and downstream pathways are frequently associated with cancer.

Although typically studied for their effects on the amplitude of tonic signaling, cancer mutations can disrupt additional features of signal transmission, including its kinetics or sensitivity, which may alter the cells’ ability to respond to extracellular signals appropriately^11–13^. One example is the EML4-ALK fusion oncoprotein^14,15^, which drives constitutive activation of ERK signaling but simultaneously suppresses signaling through RTKs, reducing the cell’s response to external growth factors. These disruptions can be reversed by targeted drugs, which restore responsiveness to growth factors and permit pulsatile ERK activity (Fig. 1B)^12^. Although examples of oncogene-induced changes in signal transmission continue to emerge, it is not clear to what extent cancer alterations corrupt a cell’s capacity to perceive its environment because changes in information transmission through dynamic signaling networks have not been quantified.

Shannon information theory^16^ provides a framework for the quantitative assessment of the rate at which information can be transferred through a communication channel, such as a signaling pathway. While *mutual information* measures the information about the input signal that can be inferred from the observed response, the *information transmission rate* (or *bitrate*) reflects the amount of information transferred through the channel per unit time. The maximum rate at which information can be transmitted through a channel using the best encoding is called the *channel capacity*. Early studies used mutual information in population-level measurements to find that the response amplitude in several important signaling pathways, including the RTK/ERK pathway, transmits only approximately 1 bit of information about the strength of a stimulus, indicating an all-or-nothing response^17,18^. Subsequent works showed that slightly more information can be transmitted when accounting for the temporal pattern of the response^19^. A recent study examined repetitiveness of ERK activation in single cells and found that cells can transmit more than 2 bits of information^20^, which could not be detected at the population level due to phenotypic diversity between individual cells^21–25^. The information transmission rate is an appropriate measure in systems where repeated use of a channel rather than a one-time decision is important because it accounts for both the fidelity of signal transmission and its temporal resolution. In such systems, information may be encoded in the intervals between signaling pulses. Recently, we showed that such encoding allows transmission of at least 6–8 bit/h through the RTK/ERK pathway^25^, exceeding earlier theoretical estimates^26^.

In this study, we apply Shannon information theory together with optogenetics and live-cell imaging to quantify changes in signal transmission resulting from the expression of the EML4-ALK fusion oncoprotein in the patient-derived STE-1 cancer cell line. We show that in this cell line, information transmission through the RTK/ERK pathway is almost entirely blocked. Treatment with the ALK inhibitor (ALKi) crizotinib restored information transmission, although to a rate still twice as low as that observed in noncancerous BEAS-2B cells (Fig. 1C). In these cells, RTK-triggered calcium/calcineurin signaling transmitted even more information than the RTK/ERK pathway, although activation of the calcineurin pathway was not observed in STE-1 cells. Our work demonstrates how information theory can provide a quantitative, functional metric to understand the oncogenic state and assess potential therapies.

## Results

### Pulsatile OptoFGFR stimulation enables estimation of information flow in the RTK/ERK pathway

To monitor and induce the activity of the RTK/ERK pathway, we engineered cells to express a fluorescent reporter of ERK activity (ERK-KTR) and an optogenetic FGF receptor (optoFGFR)^27^, which allowed us to stimulate the pathway with light at precisely selected time points (Fig. 1D). OptoFGFR is a fusion of the intracellular fragment of FGFR1 fused to the light-induced clustering module Cry2^28^. Cry2 clustering triggers activity of FGFR1 and evokes ERK pulses similar in shape and duration to those obtained by transient FGF stimulation in a microfluidic system^29^. We investigated an EML4-ALK-expressing human non-small-cell lung carcinoma cell line, STE-1, and a noncancerous cell line, BEAS-2B, as a reference. In normal cells, such as BEAS-2B cells, optoFGFR activation triggers RTK/ERK pathway signaling, culminating in ERK phosphorylation/activation and subsequent phosphorylation of its downstream targets. In STE-1 cells, EML4-ALK hijacks GRB2-SOS, leading to constitutive ERK activation and also to active suppression of transmembrane RTKs, including optoFGFR^12^. ALK inhibition relieves this suppression and restores the cells’ ability to transmit RTK signals (Fig. 1E). We monitored ERK activity using ERK-KTR, which, when phosphorylated by active ERK, translocates to the cytoplasm (Fig. 1F,G). In some cell types, including BEAS-2B cells, FGFR (and optoFGFR) also triggers calcium signaling^27^, which leads to the activation of the phosphatase calcineurin (Fig. 1A). Because ERK-KTR is engineered from a natural substrate for calcineurin (ELK-1), calcineurin can dephosphorylate and inactivate ERK-KTR, driving its nuclear localization^30^. To disambiguate signaling via the RTK/ERK and RTK/calcineurin pathways, we performed experiments in the presence or absence of inhibitors of MEK (trametinib, MEKi) and/or calcineurin (cyclosporine A, CALCi; Fig. 1F). In a typical experiment, 100 ms light pulses were administered at prespecified intervals (see below), and ERK-KTR fluorescence (Fig. 1G) was recorded every minute, resulting in single-cell trajectories of ERK-KTR nuclear intensity. In BEAS-2B cells, the preprocessed ERK-KTR trajectories (see Methods for preprocessing details) typically feature a small ‘dip’ 2 min after stimulation, resulting from calcineurin-mediated ERK-KTR dephosphorylation. This was followed by a cytoplasmic ERK-KTR translocation peak 7 min after the light pulse, which was mediated by the RTK/ERK pathway (Fig. 1H).

To measure the rate of information transmission, we stimulated STE-1 and BEAS-2B cells with a pseudorandom series of light pulses (Fig. 1I). The intervals between subsequent light pulses followed a fixed distribution (Fig. 1J), carefully chosen on the basis of preliminary experiments to ensure both a near-optimal bitrate and sampling of a broad range of intervals. Single-cell ERK-KTR trajectories enabled a probabilistic, machine learning-based reconstruction of the input signal (Fig. 1K). We used this reconstruction to compute the entropy *H*(*X* | *Y*) of the input conditioned on the observed response (i.e., information lost owing to the uncertainty of signal reconstruction). The amount of transmitted information is then given by *I*(*X*; *Y*) = *H*(*X*) − *H*(*X* | *Y*), where *H*(*X*) is the input entropy (sent information). Therefore, we computed the transmitted information rate as the input information rate minus the reconstruction entropy rate (Fig. 1L). Preliminary experiments on BEAS-2B and STE-1 cells, along with an earlier study on MCF-10A cells^25^, indicated that the effective refractory time of the RTK/ERK pathway, i.e., the interval at which the second pulse is detected with 50% probability, is approximately 8–9 min. The chosen interval distribution for our stimulus pulse trains included intervals from 5 to 35 min and had an input entropy rate of 16.4 bit/h. In principle, the input information rate could be higher for more frequent pulses, reaching 60 bit/h for pulses occurring every minute with a probability of 1/2. However, if light pulses were sent at intervals shorter than the refractory time, the cell responsiveness would deteriorate, increasing the reconstruction entropy.

### EML4-ALK blocks information transmission, which can be restored by ALK Inhibition

To estimate the reconstruction entropy (and thus the amount of information transmitted by the sequence of light pulses), we trained a multilayer perceptron (MLP with 3 layers) using all single-cell tracks longer than 3 h from both cell lines, all experimental conditions and all experimental replicates (see Methods for details). To predict the probability of a light pulse at a given time (Fig. 1K), the MLP used the following features: a 7-min fragment of the ERK-KTR trajectory, the time elapsed since the previous light pulse, a measure of track variability, and information about the cell line and an optional inhibitor. The last two inputs allowed a single MLP to generalize across experiments in which cells follow qualitatively different trajectories. During training, we directly minimized the cross-entropy, which constitutes an upper bound of the conditional entropy *H*(*X* | *Y*). By subtracting the result from the input entropy *H*(*X*), we obtained a lower bound on the transmitted information *H*(*X*; *Y*). We noticed that cells with low (or very high) expression of optoFGFR had markedly lower bitrate, and therefore we excluded them from further analysis (see Methods and Supplementary Fig. S1). We used the remaining cells (without retraining the MLP) to estimate the population-averaged bitrate at which the STE-1 and BEAS-2B cells transmit information when stimulated according to the selected protocol (Fig. 1J).

**Fig. 2:**
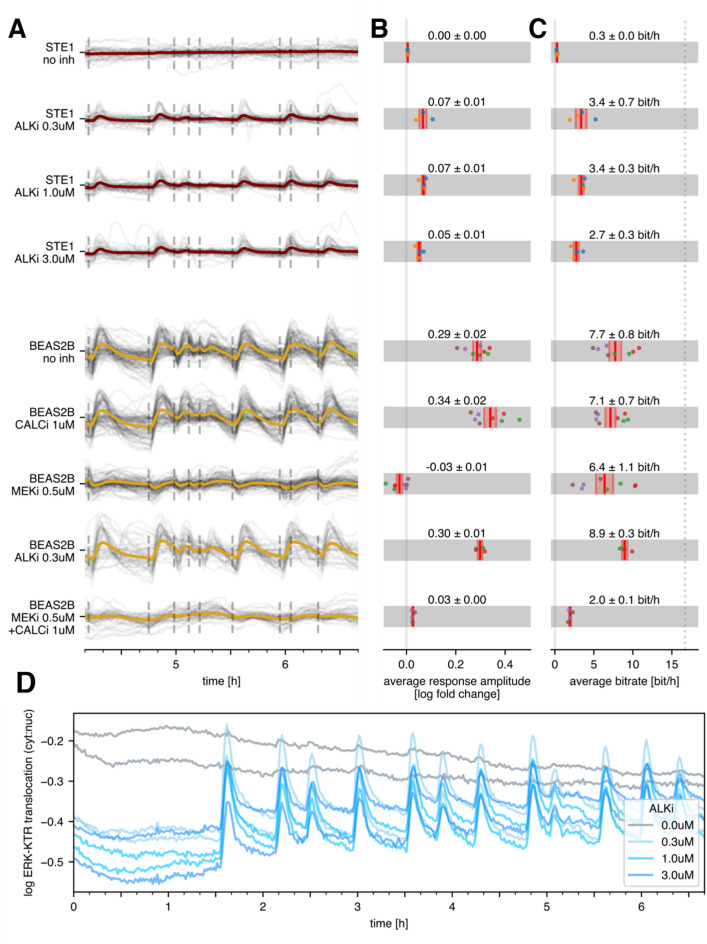
Response amplitude and bitrate in the STE-1 (cancerous) vs. BEAS-2B (noncancerous) cell lines, with or without inhibitors. (**A**) 2.5-hour fragments of ERK-KTR trajectories obtained in experiments with STE-1 and BEAS-2B cells with or without inhibitors, as indicated. The gray lines represent single-cell trajectories, whereas the colored lines represent the population average. The dashed vertical lines indicate light pulses. (**B**) Population-averaged response amplitude measured 7 min after the light pulse (the average was taken over all the cells and light pulses; see Supplementary Fig. S2 for the response amplitude definition). (**C**) Population-averaged bitrate. The dotted line shows the input information rate of the input pulse sequence. (**D**) ERK-KTR trajectory in STE-1 cells treated with various ALKi concentrations. Each line represents an average of all cells in a single technical replicate. Note that in this panel (and only here), the ERK-KTR trajectory was computed as the log-ratio of cytoplasmic and nuclear fluorescence, which allows for visualization of higher basal ERK-KTR activity in the absence of ALKi. In panels B and C, dots correspond to experimental/technical replicates; dots of the same color denote results from the same experimental replicate; bars correspond to the mean and standard error of the mean. The cell lines and stimulation protocols correspond to those in panel A.

STE-1 cancer cells were minimally responsive to light stimulation (Fig. 2A, B row 1) and thus transmitted nearly no information from the activated receptor to ERK-KTR (Fig. 2C row 1), consistent with our earlier results^12^. In contrast, when treated with ALKi, a significant fraction of cells evoked an ERK-KTR trajectory peak (Fig. 2A, B rows 2–4), which allowed for an average information transmission rate of ~3 bit/h (Fig. 2C, rows 2–4). The effect did not depend on the ALKi concentration in the tested range (0.3–3 µM), which indicates that a concentration of 0.3 µM is sufficient to maximally restore information transmission. The increase in response magnitude was due in part to a drug-induced decrease in basal signaling, which increased the dynamic range of stimulation (Fig. 2D).

We performed the same analysis for BEAS-2B cells, obtaining a 4–6 fold stronger average response and a more than twofold higher bitrate for the RTK/ERK pathway (7.2 bit/h; Fig. 2B, C, row 6), matching results obtained for MCF-10 A cells^25^. As expected, ALKi minimally affected the bitrate of BEAS-2B cells, which is consistent with their lack of ALK kinase expression (Fig. 2C, row 8 vs. row 5).

### In BEAS-2B cells, the RTK/ERK and RTK/calcineurin pathways transmit information independently

As mentioned previously, optoFGFR signals to ERK-KTR not only through ERK but also through calcineurin. The latter pathway is faster and evokes a dip (nuclear ERK-KTR translocation) in the ERK-KTR trajectory (Fig. 1H), preceding the peak (cytoplasmic ERK-KTR translocation) induced by ERK. To verify whether the high bitrate observed in BEAS-2B cells results from the MLP classifier using this dip to detect light pulses, we inhibited the RTK/calcineurin pathway with a CALCi. While CALCi eliminated the dip and slightly increased the response amplitude (Fig. 2B, row 6), it only marginally reduced the bitrate, indicating that the RTK/ERK pathway alone can transmit approximately 7.2 bit/h (Fig. 2C row 6). Similarly, after the RTK/ERK (RTK-RAS-RAF-MEK-ERK) pathway was blocked with MEKi, the RTK/calcineurin pathway was capable of transmitting approximately 6.4 bit/h. When operating simultaneously (without any inhibitors), the two pathways transmit 7.8 bit/h. As a control, we confirmed that the joint application of CALCi and MEKi prevents effective information transmission, reducing the average response amplitude tenfold and the bitrate to 2.1 bit/h.

Overall, these results show that in the noncancerous cell line, both the RTK/ERK and RTK/calcineurin pathways, separately or jointly, transmit approximately 7 bit/h when stimulated according to the chosen encoding protocol. Information transmission is fully suppressed in STE-1 cells expressing EML4-ALK but can be restored to 3 bit/h by ALKi treatment.

### In information-transmitting cells, the bitrate of ALKi-treated STE-1 cells remains half that observed in BEAS-2B cells

**Fig. 3:**
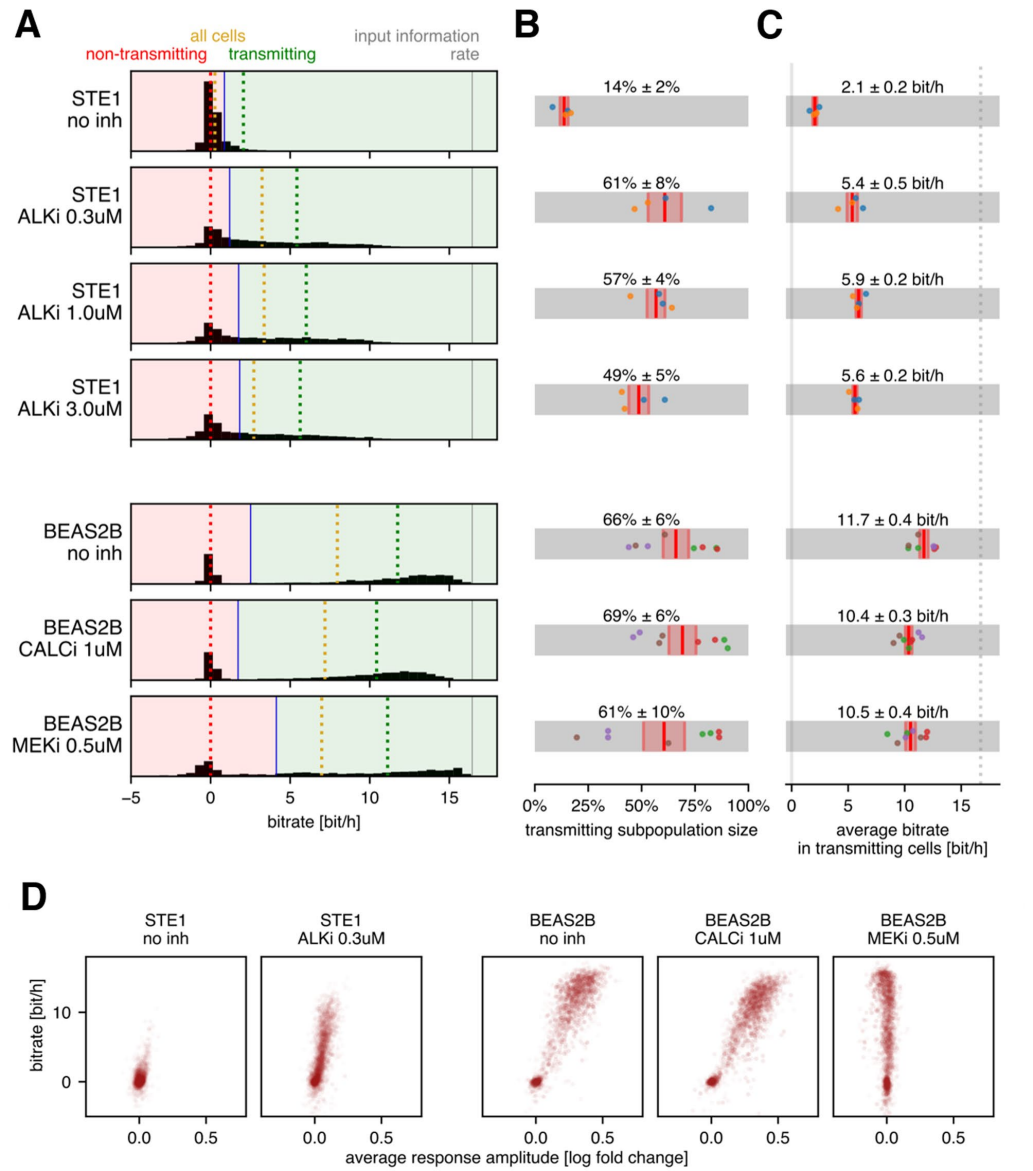
Bitrate of individual cells. (**A**) Histograms of the bitrates of individual cells; for each condition, all the biological and technical replicates are pooled. The cells are divided into transmitting and non-transmitting subpopulations so that the average bitrate in the non-transmitting subpopulation is zero. The dotted lines show the bitrate averaged over non-transmitting (red), transmitting (green), and all cells (yellow). (**B**) Fractions of transmitting cells computed for each replicate separately; average and standard error of the mean are shown. (**C**) Average bitrate in the transmitting cell subpopulation computed for each replicate separately; average and standard error of the mean are shown; the dotted line indicates the input information rate. (**D**) Bitrate vs. average response amplitude in individual cells; the dot intensity is proportional to the time for which each cell was tracked. In panels B and C, dots correspond to experimental replicates; dots of the same color denote results from the same experiment. The threshold shown in panel A (blue line) was computed for all replicates pooled, whereas in panels B and C, the threshold was chosen for each replicate separately.

To explore the differences between STE-1 and BEAS-2B cells in more detail, we assessed the heterogeneity of single-cell bitrate estimates within each cell line. The distributions (Fig. 3A) covered a wide range, from approximately −2.5 bit/h to 16.4 bit/h, the latter corresponding to the input protocol entropy rate.

Although a single cell cannot have a truly negative bitrate, our estimator can yield negative values because it is based on the cross-entropy between the input sequence and a neural network’s predictions (see Methods). Negative estimates arise when a cell responds atypically, so that the neural network (trained on all cells) systematically misinterprets its responses. In effect, the network’s predictions for such cells are worse than the prior, resulting in a net negative contribution to the overall bitrate estimate, or, equivalently, a negative single-cell bitrate estimate. For simplicity, we refer to these estimates simply as (single-cell) bitrates.

In BEAS-2B cells, the histograms of single-cell bitrate exhibit a distinct bimodal pattern: one group of cells clusters near zero, whereas the other centers at approximately 12–13 bit/h. In STE-1 cells with ALKi, the two modes overlap, with only a tiny fraction of cells transmitting more than 10 bit/h. This finding shows that even with ALKi treatment, STE-1 cells rarely transmit information at rates characteristic of transmitting BEAS-2B cells.

Formally, we split the cells into ‘non-transmitting’ and ‘transmitting’ subpopulations by setting a threshold on the histogram such that cells to the left of this threshold (‘non-transmitting’) transmit on average 0 bit/h. The average fraction of transmitting cells ranged between 60 and 70% for the BEAS-2B cells, regardless of the applied inhibitor, and between 50 and 60% for the ALKi-treated STE-1 cells (Fig. 3B). Untreated STE-1 cells consistently contained approximately 15% transmitting cells.

We noticed that both the average bitrate for the entire cell population (Fig. 2B) and the fraction of transmitting cells (Fig. 3B) were sensitive to experimental replicate-dependent factors. However, their ratio—the average bitrate within the transmitting subpopulation (Fig. 3C)—was much more stable. The transmitting subpopulation of STE-1 cells subjected to ALKi treatment consistently achieved a bitrate of approximately 5.5 bit/h, while the transmitting subpopulation of BEAS-2B cells reached 10–11 bit/h via either the RTK/ERK or RTK/calcineurin channel, and reached nearly 12 bit/h when neither channel was blocked by inhibitor.

We observed a clear correlation between the response amplitude and the bitrate in individual cells (Fig. 3D), indicating that the ‘non-transmitting’ cells are primarily non-responding. While mathematically bitrate does not depend on the response amplitude, it depends on pulse detectability, and thus the signal-to-noise ratio. Figure 3D suggests that the higher bitrate observed in BEAS-2B cells than in ALKi-treated STE-1 cells is due mainly to the higher average response amplitude, which allows for better separation of the ERK-KTR peak from background noise.

Importantly, while the difference in response amplitude between BEAS-2B cells and treated STE-1 cells is evident, its origin is not obvious. It may be associated with cancer- or drug-induced changes in RTK/ERK dynamics, but also with the cell type or reporter. It may as well result from richer (2% FBS) serum used for STE-1 to prevent cell death over the 17-hour experimental period.

### Information-transmitting cells are spatially correlated

**Fig. 4:**
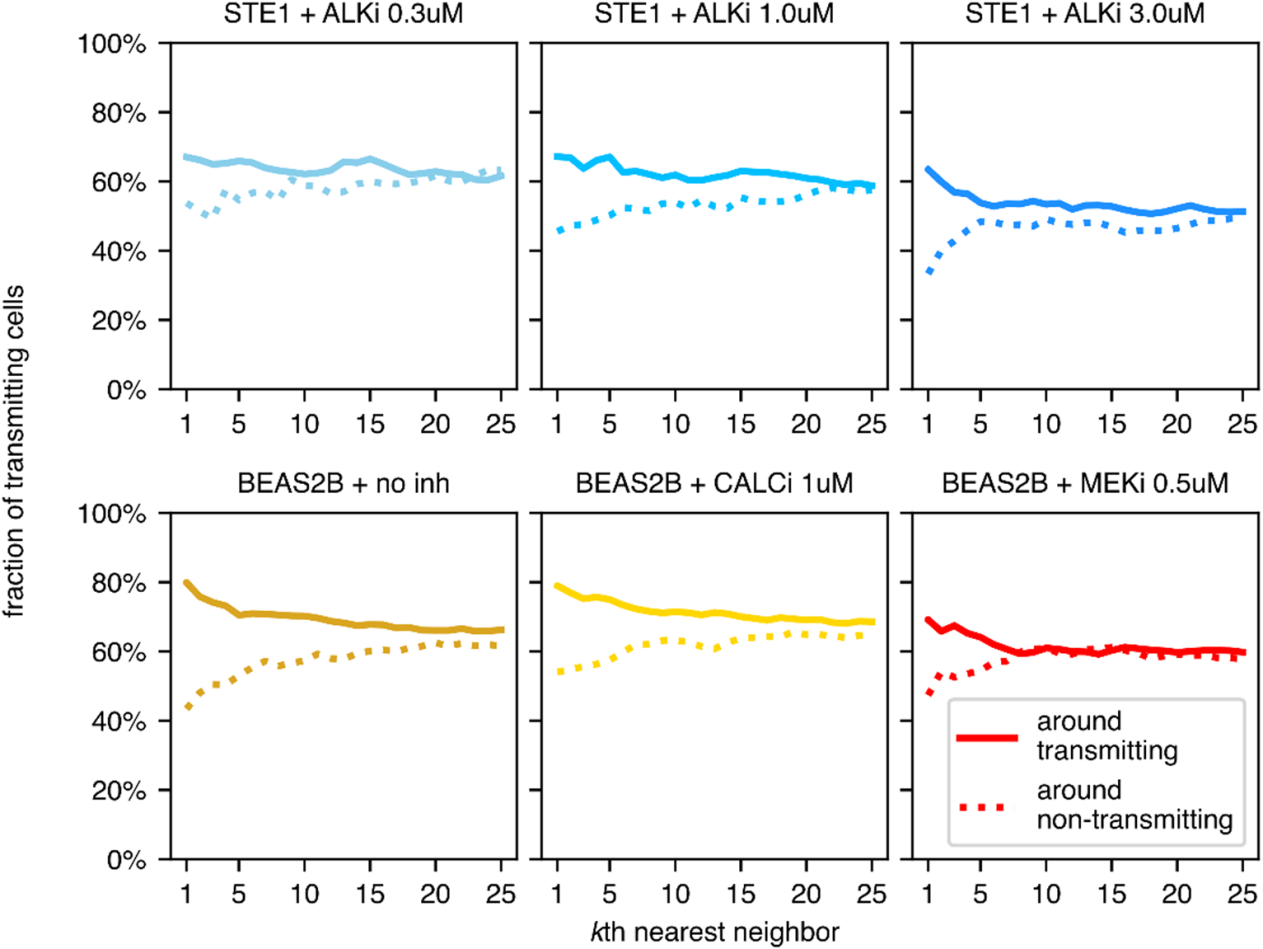
Transmitting cells are spatially correlated. Graphs show the probability that the *k*th nearest neighbor of an information-transmitting (solid) or non-transmitting (dotted) cell transmits information.

We observe that cells distinguished as information-transmitting tend to cluster. As shown in Fig. 4, the probability that the *k*th nearest neighbor of a transmitting cell transmits information is elevated with respect to neighbors of non-transmitting cells. The effect is the most visible for *k* ≲ 6. This may indicate that sister cells have a tendency to be either both transmitting or non-transmitting, although a dedicated experiment would be necessary to confirm the statement.

### Information-transmitting BEAS-2B cells tend to have both pathways functional

**Fig. 5:**
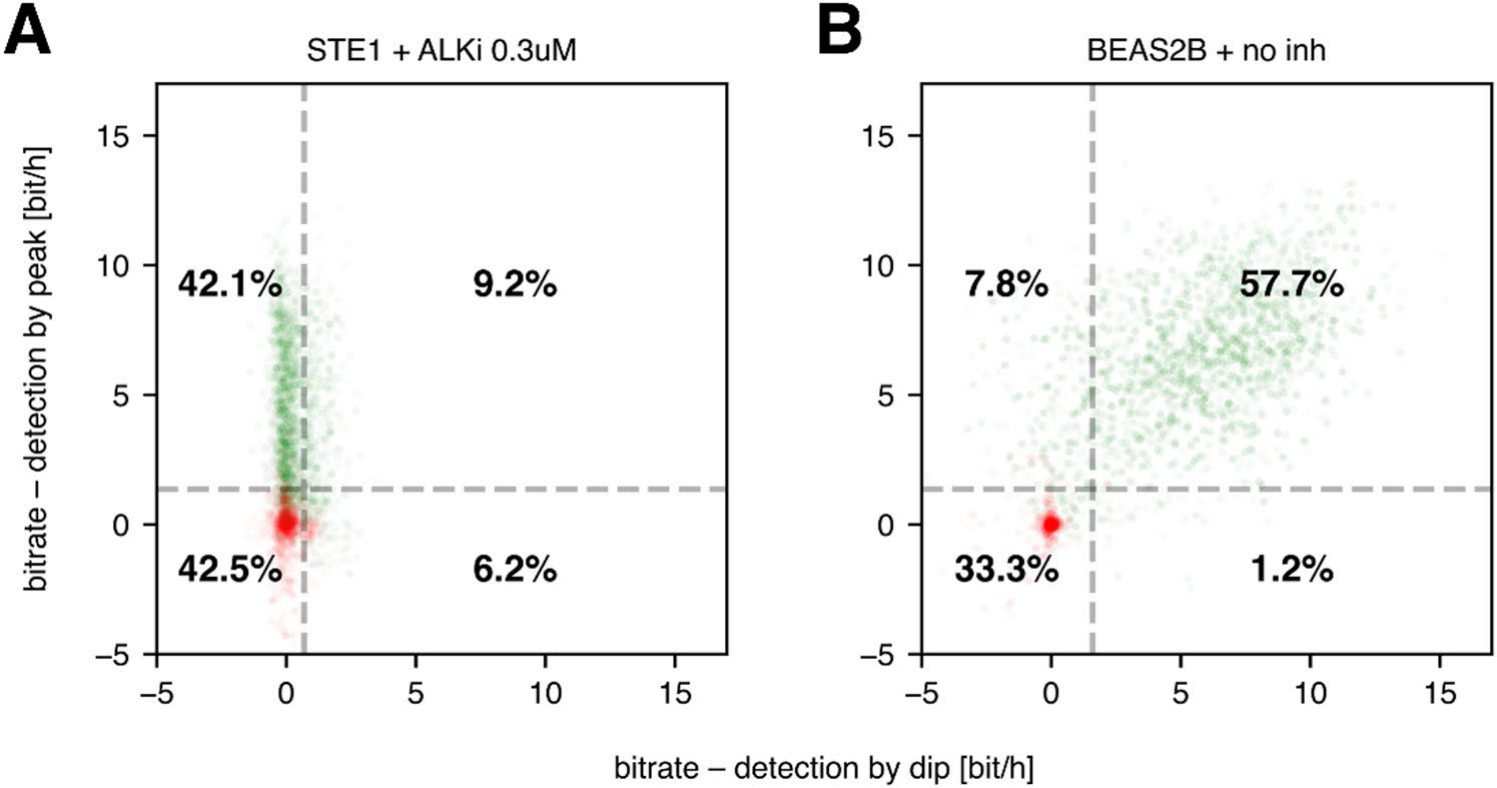
Information transmission in the RTK/calcineurin and the RTK/ERK channels. Single cell bitrates determined based on calcineurin-mediated ‘dip’ or ERK-mediated ‘peak’ only. Color indicates transmitting (green) and non-transmitting (red) cells, determined as in Fig. 3. Dashed lines separate the transmitting and non-transmitting subpopulations determined based on the ‘dip’-only or ‘peak’-only detection. Numbers indicate fraction of cells in each quarter.

The fraction of transmitting BEAS-2B cells equals 65 ± 5% regardless of the application of MEKi or CALCi (Fig. 3C). This equality suggests that the transmitting subpopulation is common and in the ‘transmitting’ cells, both the RTK/calcineurin and the RTK/ERK channels are functional. To further investigate this conjecture, we trained separate neural classifiers to recognize stimulation pulses using either the calcineurin-mediated dip or the ERK-mediated peak. This was done by altering the ERK KTR trajectory slice provided to the network: for dip-based detection, the classifier had to decide on whether a pulse occurred at time *t* based on timepoints [*t*,* …*,* t* + 2 min], while for peak-based detection, timepoints [*t* + 6 min, …, *﻿t* + 12 min] were provided. As expected, in ALKi-treated STE-1 almost no information could be retrieved by the dip-based detector (Fig. 5A), which implies that STE-1 cells did not transmit information via the calcineurin pathway. In contrast, BEAS-2B were able to respond with both pathways. As shown in Fig. 5B, there is a high correlation in information transmission between both pathways. A vast majority of cells transmit information through either both or none of the pathways with a small fraction of cells using a single channel only.

### RTK/calcineurin pathway has shorter refractory time than RTK/ERK pathway

**Fig. 6:**
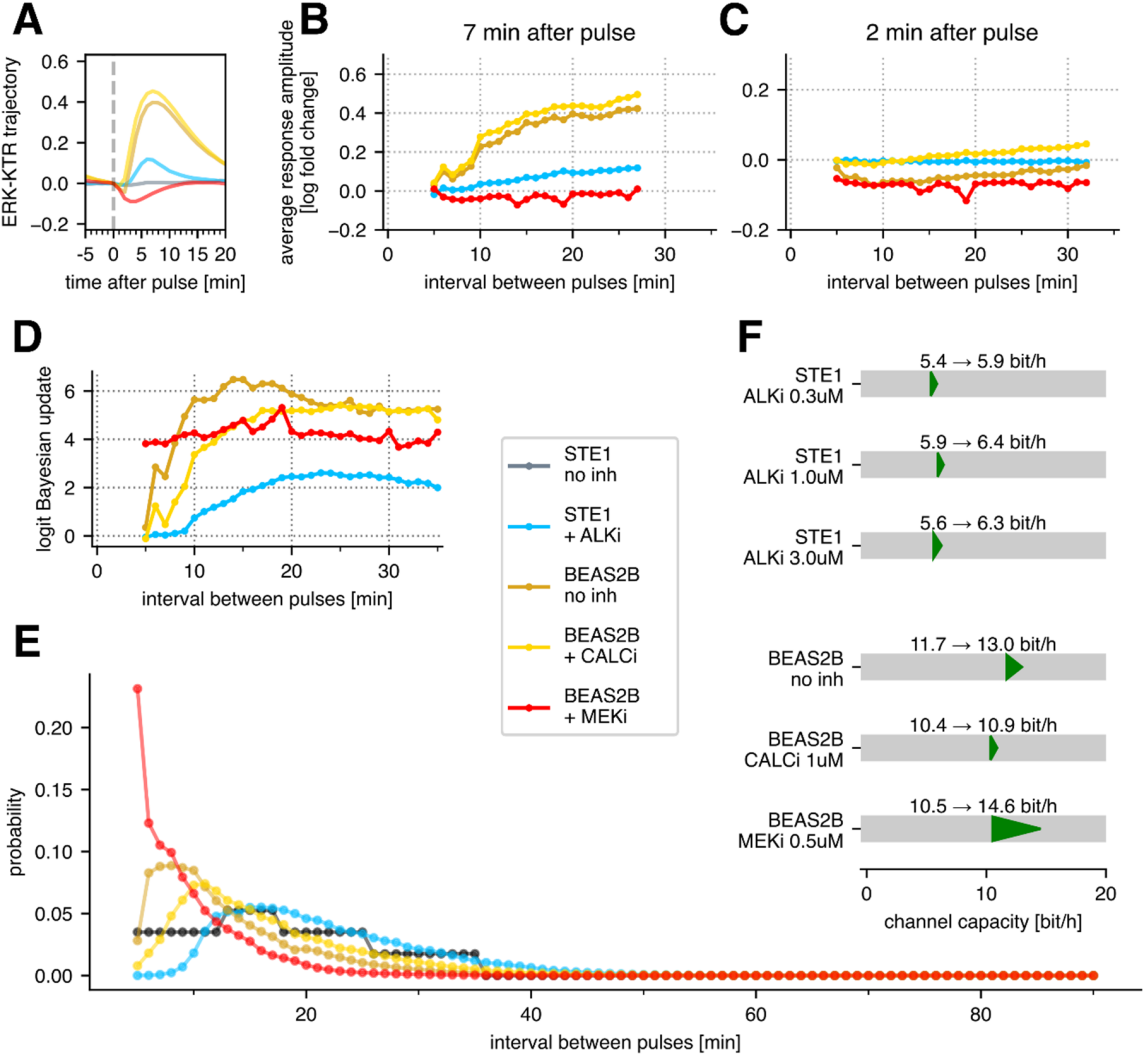
In silico protocol optimization and channel capacity estimation. In panels A–E, colors correspond to different cell lines and conditions, as indicated in the common legend. All 0.3 – 3 µM ALKi concentrations were pooled. (**A**) Population-average ERK-KTR response to a light pulse preceded by a long interval (25 min). (**B**) Average response amplitude measured 7 min after pulse, as a function of the interval preceding the light pulse. (**C**) Same as B but measured 2 min after the pulse. (**D**) The MLP classifier’s logit Bayesian update to the prior pulse probability, as a function of the interval preceding the light pulse. (**E**) Optimized input interval distribution. The black line shows the interval distribution used in the experimental protocol. (**F**) Channel capacity (i.e., bitrate achievable with the optimized input interval distribution) in transmitting cells, compared to the experimentally determined bitrates reported in Fig. 3C. Data from all experimental replicates for a given cell line and condition were pooled and analyzed jointly. Panels D–F plotted based on transmitting cells only.

The average ERK-KTR trajectory after a light pulse depends on the cell line and the applied inhibitor (Fig. 6A). In addition, the ERK-dependent response amplitude depends on the pulse interval, increasing with the time that has passed since the previous light pulse (Fig. 6B). We observed the highest response amplitude in BEAS-2B cells without inhibitors or with CALCi. In STE-1 cells without ALKi the response was close to zero (Fig. 6A). With ALKi treatment, STE-1 cells began responding to light pulses, but the response amplitude remained consistently about 5 times smaller than in BEAS-2B cells treated with CALCi or left untreated (Fig. 6B). The response amplitude in BEAS-2B cells with MEKi was negative, as the RTK/calcineurin pathway leads to ERK-KTR dephosphorylation and translocation to, rather than from, the nucleus. Moreover, the amplitude was only weakly dependent on the time since the previous light pulse, suggesting that the RTK/calcineurin pathway has a very short refractory time. When the response amplitude was measured 2 min after the light pulse (Fig. 6C), the point at which the calcineurin-induced ERK-KTR dip was the strongest, we observed that both untreated BEAS-2B and BEAS-2B with MEKi responded to intervals as short as 5 min.

Consistent with earlier observations, the certainty of pulse detection by the MLP classifier (Fig. 6D) strongly depends on the response amplitude (Fig. 6B). The certainty can be measured as the mean logit Bayesian update to the predicted light pulse probability. The logit update is zero when the classifier cannot infer any additional information from the ERK-KTR trajectory, and thus predicts the probability of a light pulse equal to the prior probability. In contrast, the logit update approaches positive or negative infinity when the pulse is predicted or excluded with 100% certainty, respectively.

We found that in untreated BEAS-2B, pulses following a 5 min interval were typically undetectable. While the response amplitude (Fig. 6B) continued to increase with longer preceding intervals, detection credibility (Fig. 6D) plateaued at intervals of ~15 min, in agreement with previous work^25^. Credibility reached half of its asymptotic value at an interval of 7–8 min, which we refer to as the effective refractory time. When calcineurin signaling was blocked with CALCi, detection of light pulses following intervals shorter than 20 min was worse than in untreated cells. This indicates that, in untreated cells, for short intervals, the network relies on both the ERK-mediated peak and the calcineurin-mediated dip to identify light pulses. The effective refractory time was increased to ~10 min. With only the RTK/calcineurin pathway active (BEAS-2B with MEKi), light pulse detection no longer depended on the interval within the tested range. This observation, consistent with Fig. 4C, indicates that the refractory time of the RTK/calcineurin pathway is shorter than 5 min. This means that the measured refractory time of the RTK/ERK channel is intrinsic to the pathway and does not result from slow reporter dynamics.

In STE-1 cells with ALKi treatment, certainty reached saturation at ~20 min intervals, as in BEAS-2B with CALCi. However, even at saturation, the neural network’s certainty remained markedly lower in STE-1 cells compared to BEAS-2B. Detection of light pulses became possible after intervals longer than 9 min, and crossed the half-asymptotic threshold at ~13 min, implying an effective refractory time ~3 min longer than in BEAS-2B with CALCi. The longer refractory time in STE-1 might suggest a discrepancy in a negative feedback mechanism between the two cell lines. However, because the response in STE-1 was weaker yet rose with similar timing as in BEAS-2B with CALCi (Fig. 6B), we suspect that the primary cause of the longer effective refractory time is an insufficient signal-to-noise ratio for short intervals in treated STE-1.

### In silico protocol optimization indicates that the RTK/ERK channel capacity is 5–15% higher than the experimentally estimated bitrate

Throughout our work, we assume that the length of intervals between subsequent pulses constitutes the ‘alphabet’ for information encoding; particular interval lengths (‘letters’) are independent, and thus, the bitrate depends only on the frequency of particular intervals. Thus far, we have estimated the bitrate assuming a fixed distribution of intervals shown in Fig. 1J. Although the interval distribution was chosen on the basis of preliminary data to yield a high bitrate for the RTK/ERK channel, it does not have to be optimal for both cell lines and, more importantly, for the RTK/calcineurin pathway. In contrast to our previous work^25^, the bitrate estimation algorithm employed in this study allowed us to use existing experimental data to make bitrate predictions for protocols that have not been experimentally tested (see Methods). Since the channel capacity is the maximum bitrate that can be achieved by optimizing the encoding protocol, this allowed us to estimate the channel capacity of the investigated pathways in silico without the need to conduct multiple experimental trials. Specifically, for each cell line and inhibitor (all STE-1 experiments with ALKi were pooled), we searched for the optimal input protocol by numerical optimization of the input interval distribution using gradient descent.

We optimized over protocols with interval lengths from 5 min to 90 min. To allow for intervals longer than 35 min (the longest interval in our dataset), we extrapolated cell responses based on the 35-min interval, which was justified by the detection certainty reaching saturation after approximately 20 min (Fig. 6D; see Methods for details). In most conditions, the cells did not respond to pulses following a 5-min interval; thus, we assumed that shorter intervals, unavailable in our experimental dataset, might be safely excluded from the optimized protocols. The only exception was BEAS-2B with MEKi, for which the data presented in Fig. 6B-D suggest that pulses following intervals shorter than 5 min may be detectable; consequently, the true channel capacity of the RTK/calcineurin pathway is likely higher than estimated here.

Most of the optimal interval distributions (Fig. 6E) follow a qualitatively similar pattern. Intervals below the detectability threshold are not sent; the interval probability rises steeply following the increase in pulse amplitude and detection certainty (Fig. 6B-D). Once the detection certainty has reached a plateau, the interval probability starts to decrease because the usage of long intervals requires more time to relay the same amount of information. The optimal trade-off between the sent information content and time budget per pulse is granted by the exponentially decreasing distribution, which we observe in all optimal protocols. In most conditions, the change in interval distribution due to optimization increased the bitrate by 5–15% (Fig. 6F). The only exception was BEAS-2B with MEKi, which experienced nearly 40% gain in the bitrate. In this case, the optimal distribution was significantly shifted toward shorter intervals, which did not affect pulse detection certainty (roughly independent of interval length; Fig. 6D) but increased the input information rate.

Overall, we found that ALKi treatment of STE-1 cells restored the RTK/ERK channel capacity to approximately 6.0–6.5 bit/h in the transmitting subpopulation. However, this value remains below the RTK/ERK channel capacity of approximately 11 bit/h estimated for BEAS-2B. The RTK/calcineurin channel in BEAS-2B cells was found to have an even higher capacity of nearly 15 bit/h (which, as mentioned before, is likely underestimated). Surprisingly, this capacity exceeds the capacity of BEAS-2B cells when both the RTK/calcineurin and the RTK/ERK pathways are unblocked (13 bit/h). This effect is probably caused by signal interference: the rise of the ERK-mediated peak counteracts the calcineurin-mediated dip (Fig. 6A) and thus hampers the dip-based detection.

## Discussion

The rate at which information is transmitted by signaling channels constrains the complexity of processes that can be regulated within a given time. High bitrates are expected for signaling pathways that regulate cellular responses to rapidly varying extracellular cues. The RTK/ERK pathway, which governs proliferation, differentiation, apoptosis, and motility, is one such pathway, and due to the nature of these processes, its malfunction is frequently associated with cancer. We note that higher bitrates are not necessarily optimal. For example, an overly-responsive cell could proliferate in response to otherwise subthreshold stimuli, leading to pathological cell growth. Rather we expect that cellular information transmission is tuned to respond to the signals—and filter the noise—of the particular tissue environment, and that disease states or molecular interventions can meaningfully alter this perceptive landscape.

In this study, using pulsatile optoFGFR stimulation, we showed that the presence of the EML4-ALK oncogene in STE-1 cells suppressed RTK/ERK signaling, reducing the pathway’s bitrate to nearly zero. However, treatment with an ALK inhibitor restored information transmission. With the inhibitor, the transmitting subpopulation of cells—comprising approximately 50–60% of all STE-1 cells—achieved an average bitrate of ~5.5 bit/h. This rate was lower than that of the RTK/ERK pathway in noncancerous BEAS-2B cells, where the transmitting subpopulation of approximately 70% reached an average bitrate of ~10.4 bit/h. The fraction of transmitting cells varied substantially across experimental replicates, which affected the average bitrate calculated across the entire population. By contrast, the average bitrate within the transmitting subpopulation (reported above) showed much lower variability and therefore can be considered a reliable metric. The reasons why the remaining cells failed to transmit information remain unclear. The immediate cause was typically a lack of response to stimulation, but we failed to attribute it to optoFGFR expression (cells with low optoFGFR expression were excluded), ERK-KTR level, nucleus size, or other factors like position within the colony or local cell density.

The lower bitrate in ALKi-treated STE-1 cells (with respect to BEAS-2B) results primarily from a lower response amplitude (higher noise to signal ratio) and a longer refractory period, leading to reduced accuracy of reconstruction. The strong correlation between the average ERK-KTR response amplitude and bitrate measured in single cells suggests that the ~5-fold weaker response strength in treated STE-1 cells may be the underlying cause of the longer measured refractory period.

In BEAS-2B cells (as in MCF-10 A cells^25^), two pathways activated by optoFGFR transmit opposing signals to ERK-KTR: the RTK/ERK pathway and the RTK/calcineurin pathway. The reporter is sensitive to calcineurin signaling because it contains an ERK docking site from the transcription factor ELK-1, which is also a substrate for dephosphorylation by calcineurin^30,31^. The RTK/calcineurin pathway thus induces rapid ERK-KTR dephosphorylation and nuclear translocation, starting 2 min after light pulse stimulation (of note, this rapid nuclear ERK-KTR translocation was not observed in STE-1). In contrast, the RTK/ERK pathway leads to ERK-KTR phosphorylation and cytoplasmic translocation, peaking approximately 7 min after the light pulse. Consequently, in BEAS-2B cells without inhibitors, ERK-KTR trajectories after a light pulse display a small ‘dip’ followed by a more pronounced ‘peak.’ By applying either a calcineurin or a MEK inhibitor, we investigated these pathways in isolation and estimated that each transmits information at a similar rate of 10–11 bit/h (again, restricting the estimation to the transmitting subpopulations). These rates are only slightly lower than the bitrate achieved without inhibitors. Our data additionally indicate that, in response to RTK stimulation, ELK-1 might undergo rapid dephosphorylation followed by phosphorylation; the biological meaning of such regulation remains to be further studied.

While bitrate is a quantity dependent on the stimulation protocol, channel capacity, i.e. bitrate in an optimal protocol, is a universal measure. The pulsatile stimulation protocol used in the study was designed to maximize the bitrate of the RTK/ERK pathway on the basis of preliminary experiments and our previous study on MCF-10 A cells^25^. It balances high information input, achieved with short intervals between stimulation pulses, against signal transmission accuracy, which deteriorates when intervals become as short as the refractory time. However, since the refractory time varies between cell lines and signaling pathways, the protocol could not be optimal simultaneously for all conditions. To address this, we developed an approach for optimizing stimulation protocols and estimating channel capacities from a single suboptimal experiment. This method works best when all intervals expected in the optimal protocol are present in the experimental protocol. Otherwise, responses to the missing intervals must be extrapolated. While the limited experiment time forced us to extrapolate data for intervals above 35 min, our data suggest that this extrapolation is generally justified. However, lack of intervals shorter than 5 min in the experimental protocol and inability to extrapolate them probably lead to an underestimation of the channel capacity of the RTK/calcineurin pathway in BEAS-2B cells. Although the optimized protocol differs from the experimental one, the estimated RTK/ERK channel capacities in BEAS-2B cells and STE-1 cells (with an ALK inhibitor) are only 5–15% higher than the experimentally measured bitrates, and equal 13 bit/h and ~6.5 bit/h, respectively. This shows that the near-optimal bitrate can be achieved by a relatively broad family of protocols. For the RTK/calcineurin pathway, the bitrate increase is much greater – approximately 40%. The high RTK/calcineurin channel capacity (~ 15 bit/h) results from its short refractory time, allowing transmission of sequences with short intervals. As mentioned, this estimate should be considered a lower bound, as even higher bitrates might be achieved with stimulation intervals shorter than 5 min. Indeed, calcium signaling plays a role in regulating rapid cellular processes, such as actin remodeling during cell movement or fertilization^32–34^.

We emphasize that all bitrates and channel capacities reported in this paper quantify information transmission from optoFGFR to ERK-KTR and thus represent lower bounds for the information reaching the upstream proteins (ERK and calcineurin). Direct measurements of ERK or calcineurin activity could yield higher values, though such measurements are currently not possible for time-resolved measurements in single cells. The measured refractory time for the RTK/ERK pathway is much longer than that of the RTK/calcineurin pathway, and therefore cannot be limited by slow ERK-KTR dynamics. Because ERK-KTR was designed based on an ERK-dependent transcription factor (ELK-1), the reported values can also be interpreted as an estimate of information available to ERK-dependent transcription factors. Some information may have been lost during processing by the neural classifier; however, the effect is likely negligible, as altering the network architecture did not improve prediction accuracy. Bitrates may also be underestimated due to the finite temporal resolution of stimulation and measurement. Nevertheless, as shown in Supplementary Fig. S5, even with the current 1-minute resolution, the network could not reliably distinguish the absence of a pulse at the time point immediately preceding stimulation. Therefore, encoding protocols with higher than 1-min temporal resolution is unlikely to increase the bitrate substantially.

In summary, we developed a machine learning-based method to analyze information flow in signaling channels and its changes as a function of an oncogenic state. We found high information capacities that confirm that ERK and calcineurin pathways can efficiently transmit signals from receptors to effector proteins. Low bitrate in EML4-ALK-positive cells highlights the impaired transmission in cancer cells, which can be at least partially restored by drug treatment. Further studies will address whether such high bitrates are fully utilized in physiological responses, whether their suppression plays a role in cancer development, and whether drug-induced relief of suppression plays an important role during therapy, for example, in drug resistance.

## Methods

### Materials

#### Cell lines

BEAS-2B cells were purchased from ATCC. STE-1 cells were a gift from Trever Bivona lab, UCSF.

#### Inhibitors

Crizotinib (Sigma-Aldrich, PZ0191) was used as an ALK inhibitor (ALKi).

Trametinib (Selleckchem, GSK1120212) was used as a MEK inhibitor (MEKi).

Cyclosporin A (Thermo Fisher Scientific, AAJ6319103) was used as a calcineurin inhibitor (CALCi).

#### Plasmid expression

The optoFGFR-encoding plasmid CLPIT Myr-mEGFP-FGFR(ICD)-Cry2 was cloned by substitution of mCherry with mEGFP in CLPIT Myr-mCherry-FGFR(ICD)-Cry2^12^.

The cells were infected sequentially with pLentiPGK DEST-H2B-iRFP670 (Addgene #90237) for nuclear imaging, pHR ErkKTR-mRuby2 or pHR ErkKTR-mCherry (STE-1 or BEAS-2B, respectively) for ERK activity measurement and CLPIT Myr-mEGFP-FGFR(ICD)-Cry2 for stimulation.

Infected cells were sorted twice to enrich for high expression of all 3 markers.

### Experimental procedure

#### Cell culture

The cells were cultured at 37 °C and 5% CO_2_ in RPMI-1640 growth medium supplemented with 10% fetal bovine serum (FBS) and 1% penicillin/streptomycin (P/S). For the experiments, cells (STE-1, 1.5 × 10^4^; BEAS-2B, 5 × 10^3^) were seeded 48 h before the experiment in 96-well plates (Cellvis) coated with fibronectin (MilliporeSigma, FC01010MG) diluted to 10 µg/mL in PBS. The media were replaced 16 h before the experiment with RPMI-1640 w/o phenol red, either serum-free or containing 4% FBS (for BEAS-2B or STE-1, respectively). Upon the initiation of the experiment, an equal volume of serum-free medium with supplements or DMSO was added to reach the indicated concentrations.

#### Imaging

Live-cell imaging was performed using a Nikon Ti2-E microscope equipped with a Yokagawa CSU-W1 spinning disk, 405/488/561/640 nm laser lines, an sCMOS camera (Photometrics), and a motorized stage. The cells were maintained at 37 °C and 5% CO_2_ in an environmental chamber (Okolabs). The cells were imaged every 60 s for 90 min without optoFGFR stimulation, followed by the stimulation protocol described in the next subsection. The 561 nm line was used for ERK-KTR-mRuby2 and ERK-KTR-mCherry imaging (80% laser power; 150 ms), and the 640 nm line was used for H2B-iRFP imaging (80% laser power; 200 ms). OptoFGFR was stimulated and imaged with 488 nm (100% laser power, i.e., 1.15 W/cm^2^; 100 ms) at the indicated time points.

#### Stimulation sequence

In all the experiments, the cells were stimulated with an identical sequence of light pulses (Supplementary Table S1). The sequence was constructed as follows. First, on the basis of preliminary data and earlier results^25^, we decided that the intervals between subsequent light pulses should approximately follow a Gamma distribution with shape *α* = 4 and scale *θ* = 5 min. This choice was expected to be near-optimal in terms of bitrate and broad enough to probe various interval lengths. The actual number of intervals of a particular length in the sequence (Fig. 1I) was chosen to best reflect this distribution given the time budget (~ 17 h). Minor adjustments were made to ensure that each short interval length occurred at least twice. The intervals were ordered in such a way that the occurrences of a particular interval length were preceded by intervals maximally representative of the assumed distribution. For example, there were three intervals of length 13 min in the sequence; they were preceded by intervals of lengths 7, 17, and 28 min, which were close to the 1 st, 2nd, and 3rd quartiles of the assumed distribution, respectively. This was important for a fair comparison between interval lengths (Fig. 6) because a pulse following a short interval evokes a stronger response if the interval before it was also short; consequently, if all intervals of a particular length were preceded by short intervals, the detectability of this interval length would be overestimated.

### Image processing

#### Cell tracking

The cell nuclei were detected based on H2B-iRFP fluorescence and tracked using our in-house software ShuttleTracker. Tracks shorter than 3 h were removed from further analysis.

#### Track standardization

We estimated the background intensity in each channel as the mode of the pixel intensity (a common value was chosen across all frames and replicates with equal microscope settings) and subtracted it from the image. For each cell in a frame, we measured the average nuclear intensity in the ERK-KTR channel and normalized it with the average intensity over the whole frame. These two steps were necessary to eliminate global changes in mRuby2 fluorescence arising in response to the 488 nm light used for optoFGFR stimulation and to compensate for illumination differences across frames and replicates. The time course of the negative logarithm of the obtained value is referred to as a normalized *ERK-KTR trajectory*. We used this value instead of the typically used cytoplasmic-to-nuclear ratio to avoid noise associated with inaccurate cytoplasm detection. For a clearer graphical presentation in Fig. 2A, the single-cell ERK-KTR trajectories were normalized by subtracting the average value in each track.

#### Preselection of optoFGFR-expressing cells

We sorted the tracked nuclei in each replicate based on the mean optoFGFR fluorescence intensity at the time points at which the optoFGFR was stimulated and discarded tracks outside the range [*µ*, *µ* + 3*σ*] (BEAS-2B) or [*µ* − 0.5*σ*, *µ* + 2*σ*] (STE-1), where *µ* and *σ* denote the track-length-weighted average and standard deviation across all tracks in the particular replicate (see Supplementary Fig. S1).

#### Response amplitude and responsiveness

For each track and light pulse, we computed the *response amplitude* as the log-ratio of the ERK-KTR trajectory 7 min after the pulse and at the pulse. To account for the influence of the previous pulse, we normalized it by subtracting the average log-ratio of the ERK-KTR trajectory (*L* + 7 min) and *L* after the pulse, where *L* is the interval between this pulse and the previous pulse. The average was taken across all tracks and all pulses followed by at least (*L* + 7 min)-long intervals (Supplementary Fig. S2).

We also computed the cell’s *responsiveness* as the standard deviation across all time points (not just time points with pulses) of the log fold change in translocation over 7 min. This measure was independent of pulse timing and, as such, could be used as a hint in automated pulse detection, as described below.

### Bitrate computation

#### Derivation of the bitrate lower bound

We consider random sequences of light pulses that may occur in whole minutes. These sequences are encoded as *X* = *X*_1_, …, *X*_*N*,_ where *X*_*k*_ = 1 if there was a pulse sent at the *k*th minute and *X*_*k*_ = 0 otherwise. We note that *X*_*k*_ need not be independent. For each cell, we denote the sequence of responses (ERK-KTR trajectories) as *Y* = *Y*_1_, …, *Y*_*N*_. The mutual information between these two sequences can be computed as:

1 $$\begin{aligned} I\left( {X;Y} \right) & = H\left( X \right) - H\left( X\:\middle| \:Y \right) \\ & = \sum\limits_{k} {\left[ {H\left( X_{k} \:\middle| \:X_{{1\dots k - 1}}  \right) - H\left( X_{k} \:\middle| \:X_{{1\dots k - 1}} ,Y \right)} \right]} \\ & = - \sum\limits_{k} {\left[ {{\mathbb{E}}\log p\left( X_{k}\:\middle|\: X_{{1\dots k - 1}}  \right) -{\mathbb{E}}\log p\left( X_{k} \:\middle|\: X_{{1\dots k - 1}} ,Y \right)} \right]} \\ & = - \sum\limits_{k} {{\mathbb{E}}\:\left[ {\log p\left( X_{k} \:\middle|\:X_{{1\dots k - 1}}  \right) - \log p\left( X_{k}\:\middle|\:X_{{1\dots k - 1}} ,Y \right)} \right]} \\ \end{aligned}$$

For independent *X*_*k*_, (*X*_*k*_ | *X*_1…*k*−1_) = *H*(*X*_*k*_). In this case, the intervals between pulses follow a geometric distribution. However, we consider arbitrary interval distributions, and thus (*X*_*k*_ | *X*_1…*k* −1_) ≤ *H*(*X*_*k*_).

For a sufficiently large number of time points, *N*, the bitrate (or transmitted information per time point), b(*X*; *Y*), can be estimated as:

2 $$\begin{aligned} b\left(X;Y\right)&=I\left(X;Y\right)/\:N \\ &=-\frac{1}{N}\sum\limits_{k}\mathbb{\:}\mathbb{E}\mathbb{\:}\left[\log p\left({X}_{k}\:\middle|\:{X}_{1\dots k-1}\right)-\log p\left({X}_{k}\:\middle|\:{X}_{1\dots k-1},Y\right)\right] \\ &=-\mathbb{\:}\mathbb{E}\mathbb{\:}\left[\log p\left({X}_{k}\:\middle|\:{X}_{1\dots k-1}\right)-\log p\left({X}_{k}\:\middle|\:{X}_{1\dots k-1},Y\right)\right]\end{aligned}$$

where in the last equation, the expected value is also calculated over all time points *k* = 1, …, *N*. Thus, b(*X*; *Y*) is equal to the expected difference between the surprisal of *X*_*k*_ conditioned on all previous pulses, −log p(*X*_*k*_ | *X*_1…*k*−1_), and the surprisal of *X*_*k*_ conditioned on all previous pulses and additionally the full response Y, −log p(*X*_*k*_ | *X*_1…*k*−1_, *Y*). This can be thought of as the expected reduction in surprisal from learning *Y*.

To estimate b(*X*; *Y*), we first note that the probability p(*X*_*k*_ | *X*_1…*k*−1_) depends only on the assumed encoding protocol. We restrict our analysis to protocols for which the probability of a pulse at time point *k* depends only on the time that elapsed since the previous pulse, *Last*_k_ (*Last*_k_ = *l* if and only if *X*_*k*−*l*_ = 1 and *X*_*k*'_ = 0 for all *k'* in {*k* − *l* + 1, …, *k* − 1}). Thus:

3 $$\:p\left({X}_{k}\:\middle|\:{X}_{1\dots k-1}\right)=p\left({X}_{k}\:\middle|\:{Last}_{k}\right)$$

The posterior probability p(*X*_*k*_ | *X*_1…*k*−1_, *Y*) cannot be directly computed. Instead, we estimate it by training a neural network p_θ_ (with weights θ) to predict *X*_*k*_. In principle, the neural network could use the complete stimulation history *X*_1…*k*−1_ and the full response *Y* to predict *X*_*k*_. However, we simplify this by reducing *X*_1…*k*−1_ to *Last*_*k*_, and *Y* to a slice *Y*_*k*…*k*+*r*_ (of length *r* + 1 = 7, starting at time point *k*) and a scalar measure of cell responsiveness *S* = Std(*Y*_*k*_ − *Y*_*k*+7_). This reduction is necessary, as the network is trained and tested on the same sequence of pulses; thus, the stimulation history *X*_1…*k*−1_, as well as the full response trajectory *Y*, can be used to infer the timepoint in the experiment (*k*) and consequently reveal X_k_. By providing the network with *Last*_k_ instead of *X*_1…*k*−1_ and the slice *Y*_*k*…*k*+*r*_ instead of *Y*, we prevent this leak. In summary, we approximate the posterior probability as:

4 $$\begin{aligned} & p\left({X}_{k}\:\middle|\:{X}_{1\dots k-1},Y\right) \\  \approx\:&p\left({X}_{k}\:\middle|\:{Last}_{k},\:{Y}_{k\dots k+r},S\right) \\  \approx\:&{p}_{\theta}\left({X}_{k}\:\middle|\:{Last}_{k},\:{Y}_{k\dots\:k+r},S\right)\end{aligned}$$

This approximation results in an upper bound on the conditional entropy:

5 $$\begin{aligned}H\left({X}_{k}|{X}_{1 \dots k - 1},Y\right)& \le\:H\left({X}_{k}\:\middle|\:{Last}_{k},\:{Y}_{k \dots k + r},S\right) \\ &\le\:-\mathbb{E}\mathbb{\:}\left[\log{p_{\theta}}\left({X}_{k}\:\middle|\:{Last}_{k},\:{Y}_{k \dots k+r},S\right)\right]\end{aligned}$$

where the expectation is still computed over the true distribution of *X*, *Y*, and time points *k*. The first inequality follows from the data processing inequality, and the second reflects the fact that the cross-entropy between p and p_θ_ is an upper bound on the entropy of p. Thus, by substituting (5) back into formula (2), we obtain a lower bound on the bitrate:

6 $$\:b\left(X;Y\right)\ge\:-\mathbb{E}\left[\log{p\left({X}_{k}\:\middle|\:{Last}_{k}\right)}-\log{{p}_{\theta}\left({X}_{k}\:\middle|\:{Last}_{k},\:{Y}_{k\dots k+r},S\right)}\right]$$

The bound is tight, assuming that the network correctly predicts the true posterior probability (both approximations in Eq. (4) are equalities). We estimate the bitrate by averaging over all cells *j* and timepoints *k* available in our dataset:

7 $$\:b=-\frac{1}{D}\sum\limits_{j,k}\left[\log {p\left({x}_{k}\:\middle|\:{last}_{k}\right)}-\log{{p}_{\theta}\left({x}_{k}\:\middle|\:{last}_{k},\:{y}_{k \dots k+r}^{j},\:{s}^{j}\right)}\right]$$

where *D* is the number of data points (*j*, *k*), and small letter symbols *x*_*k*_ and *last*_k_ denote values from distributions *X*_*k*_ and *Last*_k_.

#### Single-cell bitrates

We define the bitrate of a given cell track *j* as:

8 $$\begin{gathered}{b}_{j}=-{\mathbb{E}}_{k}\left[\log{{p}_{\theta}\left({X}_{k}\:\middle|\:{Last}_{k}\right)}-\log{{p}_{\theta}\left({X}_{k}\:\middle|\:{Last}_{k},\:{Y}_{k\dots k+r},S\right)}\right] \\=-\frac{1}{{w}_{j}}\sum\limits_{k}\left[\log{p\left({x}_{k}\:\middle|\:{last}_{k}\right)}-\log{{p}_{\theta}\left({x}_{k}\:\middle|\:{last}_{k},\:{y}_{k\dots k+r}^{j},\:{s}^{j}\right)}\right]\end{gathered}$$

where *w*_*j*_ is the number of timepoints available for cell track *j*. Note that since we use a model trained on all tracks, if a cell responds aberrantly, it can cause the model to make worse predictions than the prior, resulting in a negative bitrate of that track. The overall bitrate can be expressed as an average over the individual cell track bitrates (weighted by cell track length):

9 $$\begin{aligned}&b=-\frac{1}{D}\sum\limits_{j,k}\left[\log{p\left({x}_{k}\:\middle|\:{last}_{k}\right)}-\log{{p}_{\theta}\left({x}_{k}\:\middle|\:{last}_{k},\:{y}_{k\dots k+r}^{j},\:{s}^{j}\right)}\right] \\ &=-\frac{1}{D}\sum\limits_{j}{w}_{j}\left(\frac{1}{{w}_{j}}\sum\limits_{k}\left[\log{p\left({x}_{k}\:\middle|\:{last}_{k}\right)}-\log{{p}_{\theta}\left({x}_{k}\:\middle|\:{last}_{k},\:{y}_{k\dots k+r}^{j},\:{s}^{j}\right)}\right]\right)\\ &=\frac{1}{D}\sum\limits_{j}{w}_{j}{b}_{j} =\frac{\sum_{j}{w}_{j}{b}_{j}}{\sum_{j}{w}_{j}}\end{aligned}$$

Since *w*_*j*_ is the number of timepoints available for cell *j*, *D* = Σ_*j*_ *w*_*j*_.

#### Fraction of transmitting cells

The fraction of transmitting cells is determined by sorting the single-cell bitrates in ascending order and taking the longest prefix that averages (weighted by cell track length) to a negative value. The cells in the prefix are called non-transmitting, whereas the rest constitute the transmitting subpopulation.

### Network training procedure

#### Data sampling

The network is trained to minimize the cross-entropy loss function using stochastic gradient descent:

10 $$\:\mathcal{L}=-\mathbb{E}\log{p}_{\theta}\left({X}_{k}\:\middle|\:{Last}_{k},\:{Y}_{k\dots k+r},S\right)$$

At each step, the expected value is computed by averaging over a mini-batch of 10^4^ data points (x_k_, *last*_k_, *y*^*j*^_*k*…*k*+*r*_, *s*^*j*^) randomly drawn from the dataset. In general, the network predictions *p*_θ_ depend on the assumed input protocol (interval distribution). Although throughout the paper, we use a network trained according to the experimentally tested interval distribution, we designed the sampling procedure to allow for training and evaluation using an arbitrary protocol. We group all the data points by *interval*_k_ (the length of the interval containing the data point) and *last*_k_ (the time elapsed since the previous pulse). We first sample pairs (*interval*_k_, *last*_k_) according to their distribution in the assumed protocol, and then randomly retrieve data points from the corresponding groups in the experimental dataset to form mini-batches.

**Table:** 

Procedure:
1. Group dataset by (*interval*_k_, *last*_k_). 2. Sample pairs of (*interval*_k_, *last*_k_) from the joint distribution of the assumed protocol. 3. Select random samples from the dataset for each (*interval*_k_, *last*_k_) pair.

#### Multiple experiments

Throughout the paper, we use a single network trained on all the experiments. We allow the network to adapt to different experimental settings by supplying additional inputs:

11 $$\:\mathcal{L}=-\mathbb{E}\log{p}_{\theta}\left({X}_{k}\:\middle|\:{Last}_{k},\:{Y}_{k\dots k+r},S,CellLine,\:Inhibitor\right)$$

where *CellLine* is 0 for STE1 and 1 for BEAS-2B, and *Inhibitor* is a triplet of real numbers providing concentrations of 3 inhibitors {*ALKi*,* MEKi*,* CALCi*}. We then performed leave-one-out cross-validation on the experimental replicates (see Supplementary Fig. S3). Since the change in bitrate estimate after exclusion of any single replicate is negligible, we concluded that the network does not overfit to individual replicates and that we may use a single network trained on all available data.

#### Network architecture

The network inputs are log(*last*_k_), *y*^*j*^_*k*…*k*+*r*_, *s*^*j*^, *cellLine*, and *inhibitor* (3-dim). The trajectory slice *y*^*j*^_*k*…*k*+*r*_ is replaced with discrete differences Δ*y*^*j*^_*k*+1…*k*+*r*_, where Δ*y*^*j*^_*k'*_ = *y*^*j*^_*k*'_ − *y*^*j*^_*k*'−1_, to abstract from the base level of ERK-KTR expression in individual cells. We chose to use the slice of *r* = 6 discrete differences, as its further extension provides no substantial increase in bitrate (see Supplementary Fig. S4). To detect pulses based on the ‘dip’ or ’peak’-only (Fig. 5), we use, respectively, slices *y*^*j*^_*k*…*k*+*2*_ and *y*^*j*^_*k+6*…*k*+*12*_. All the inputs are concatenated, resulting in a feature vector of length (*r* + 6). The features are standardized across the dataset to have zero mean and a standard deviation of *σ* = 1, and passed to a three-layer perceptron (MLP) with hidden layer sizes of (40, 20) and leaky ReLU activations. The MLP computes a logit (Bayesian) update *u*_Bayes_ to the prior probability *p*(*X*_*k*_ = 1 | *Last*_k_):

12 $$\:{u}_{Bayes}=MLP\left(\log {last}_{k},\varDelta\:{y}_{k+1\dots k+r}^{j},\:{s}^{j},\:cellLine,\:inhibitor\right)\:$$

This update is then used to compute the posterior (logit) probability of X_k_ = 1:

13 $${\mathrm {logit}}\:{p}_{\theta}\left({X}_{k}=1\:\middle|\:{last}_{k},\:{y}_{k\dots k+r}^{j},\:{s}^{j},\:cellLine,\:inhibitor\right)={u}_{Bayes}+{\mathrm {logit}}\:p\left({X}_{k}=1\:\middle|\:{last}_{k}\right)$$

This approach allows the network to generalize more effectively across protocols with different priors *p*(*X*_*k*_ = 1 | *Last*_k_), simplifying protocol optimization.

Supplementary Fig. S5 illustrates the mean updates u_Bayes_ generated by the MLP, grouped by (*interval*_k_, *last*_k_) for STE-1 with ALKi. A positive value indicates that the network consistently predicts a higher pulse probability than the prior probability—this is the case along the diagonal (*interval*_k_ = *last*_k_), corresponding to timepoints with actual pulses (*X*_*k*_ = 1). A value below zero indicates that the network predicts a lower pulse probability than the prior. A value close to zero implies that the network cannot improve upon the prior probability. As a result, no information is gained. This behavior is observed for *last*_k_ ≤ 10, where cells have not yet recovered from a previous pulse and cannot respond to a new pulse (see also Fig. 6B). Consequently, their ERK-KTR trajectory carries no information about a potential new pulse. For *last*_k_ > 10, off-diagonal terms (where *X*_*k*_ = 0) are generally negative. For such *last*_k_, a pulse would evoke a noticeable ERK-KTR trajectory change; thus, the absence of such a change leads the network to infer that no pulse has occurred and predict a probability lower than the prior probability. Importantly, these negative predictions also contribute to the total information transmitted and thus the bitrate. Finally, we note that entries immediately next to the diagonal are also close to zero. This suggests that the network sees a response to the pulse but is not perfectly sure of the exact timing of the pulse.

#### Protocol optimization

To optimize the protocol, we want to directly *maximize* the bitrate estimate:

14 $$\:\mathcal{L}=-\mathbb{E}\left[\log p\left({X}_{k}\:\middle|\:{Last}_{k}\right)-\log{p}_{\theta}\left({X}_{k}\:\middle|\:{Last}_{k},\:{Y}_{k \dots k+r},S\right)\right]$$

This introduces several challenges. First, during training, the protocol’s interval probabilities are optimized via gradient descent. However, the expected value is computed by sampling according to the protocol; thus, we need to compute a derivative of the form  ∇_ϕ_${\mathbb{E}}$_z~Z(ϕ)_ f(z) for some distribution *Z* parameterized by ϕ. This is accomplished by rewriting the expected value using a frozen distribution with importance weights:

15 $$\:{\mathbb{E}}_{z\sim Z\left(\varphi\right)}f\left(z\right)={\left.\left[{\mathbb{E}}_{z\sim Z\left({\varphi}^{{\prime}}\right)}f\left(z\right)\frac{{p}_{\varphi}\left(z\right)}{{p}_{{\varphi}^{{\prime}}}\left(z\right)}\right]\right|}_{{\varphi}^{{\prime}}=\:\varphi}\:$$

Since the sampled distribution no longer depends on ϕ, the gradient ∇_ϕ_ may be calculated as follows:

16 $$\begin{aligned} &{\nabla}_{\varphi}{\left.\left[{\mathbb{E}}_{z\sim Z\left({\varphi}^{{\prime}}\right)}f\left(z\right)\frac{{p}_{\varphi}\left(z\right)}{{p}_{{\varphi}^{{\prime}}}\left(z\right)}\right]\right|}_{{\varphi}^{{\prime}}=\varphi} \\ &={\left.\left[{\mathbb{E}}_{z\sim Z\left({\varphi}^{{\prime}}\right)}{\nabla}_{\varphi}\left(f\left(z\right)\frac{{p}_{\varphi}\left(z\right)}{{p}_{{\varphi}^{{\prime}}}\left(z\right)}\right)\right]\right|}_{{\varphi}^{{\prime}}=\varphi}\end{aligned}$$

Although the importance weights *p*_ϕ_(*z*)/*p*_ϕ'_(*z*) are equal to 1, their derivatives with respect to ϕ in general differ from zero and allow us to compute the gradient. In our case, we sample from a frozen protocol and update the protocol after each gradient step is calculated.

Secondly, the optimal protocol should assign nonzero probabilities to intervals longer than 35 min, the longest interval used in our experiments. Since we observe that responses for intervals longer than 30 min are similar (and strongest), we handle longer intervals by imputing them using the available 35-minute interval from our experiments. Specifically, when a trajectory slice for a pair (*interval*_k_, *last*_k_) with *interval*_k_ > 35 min is requested, we return a slice for an imputed pair (*interval*_k_*, *last*_k_*) instead, where *interval*_k_* = 35 min and *last*_k_* is determined as follows:

- If *last*_k_ < 10 min: *last*_k_* = *last*_k_
- If *interval*_k_ − *last*_k_ < 10 min: *last*_k_* = 35 min − (*interval*_k_ − *last*_k_).
- Otherwise:

17 $$\:las{t}_{k}^{*}=10\:{\mathrm {min}}\:+\left({last}_{k}-10\:{\mathrm {min}}\right)\times\frac{35\:{\mathrm {min}}\:-20\:{\mathrm {min}}}{{interval}_{k}-20\min}$$

In this way, we keep the time since the last pulse and the time to the next pulse fixed if this time is shorter than 10 min. A graphical illustration of imputation for *interval*_*k*_ = 60 min is shown in Supplementary Fig. S6. Note that while *y*_*k*…*k*+*r*_ is imputed, the network is provided with true *last*_k_.

Furthermore, selection of the transmitting subpopulation technically depends on single-cell bitrates, and thus also depends on the protocol. We perform protocol optimization on the transmitting subpopulation determined using the experimental protocol.

Finally, the optimized protocol can exhibit large jumps in probabilities of specific intervals owing to experimental noise and the order in which intervals were tested in the experiment. To address this, we introduce a regularization term in the loss function to promote smoother protocols:

18 $$\:{\mathcal{L}}_{reg}=\mathcal{L}-\alpha\:{{\Vert\varDelta\varDelta\:ProtocolLogProbabilities \Vert}_{2}}^{2}$$

where ΔΔ*ProtocolLogProbabilities* represents the second-order differences in the interval log-probabilities, and ‖·‖_2_^2^ is the mean of squares. We found that setting = 0.003 yields noticeably smoother protocols with minimal impact on the bitrate (see Fig. 6E and Supplementary Fig. S7).

## Supplementary Information

Below is the link to the electronic supplementary material.

Supplementary Material 1

## Data Availability

Data used in the paper (time-lapse microscopy quantifications) are deposited on Zenodo (https://doi.org/10.5281/zenodo.15282876). Raw images can be obtained from Tomasz Lipniacki (tlipnia@ippt.pan.pl). Source code is available on GitHub (https://github.com/pawelnalecz/pathway-bitrate). Cell lines and plasmids generated for this study are available from Lukasz J. Bugaj (bugaj@seas.upenn.edu).
