# Supplementary material for: Quantifying cancer- and drug-induced changes in Shannon information capacity of RTK signaling
Source: Sci Rep. 2025 Nov 10;15:39340. doi: 10.1038/s41598-025-23075-y (PMC12603049; doi:10.1038/s41598-025-23075-y)
Supplement: Supplementary file 1 — Supplementary Material 1 [file 41598_2025_23075_MOESM1_ESM.pdf]

Supplementary Materials

Quantifying cancer- and drug-induced changes in Shannon information capacity of RTK signaling

Paweł Nałęcz-Jawecki[1]*, Lee Roth[2]*, Frederic Grabowski[1], Sunnie Li[2], Marek Kochańczyk[1], Lukasz J. Bugaj[2]✉, Tomasz Lipniacki[1,3]✉

[1] Institute of Fundamental Technological Research, Polish Academy of Sciences, Warsaw, 02-106, Poland

[2] Department of Bioengineering, University of Pennsylvania, Philadelphia, PA, 19104, USA

[3] Department of Statistics, Rice University, TX, 77251, USA

* These authors contributed equally.

✉ Corresponding authors: tlipnia@ippt.pan.pl (T.L.), bugaj@seas.upenn.edu (L.J.B.)

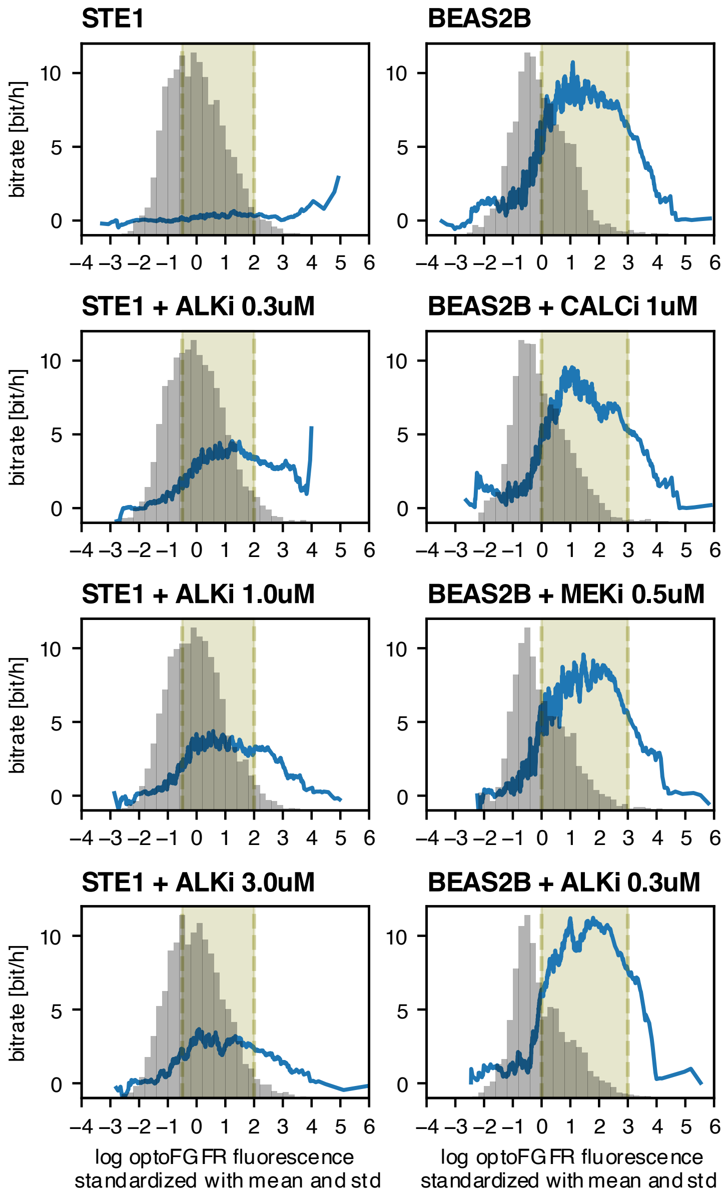

Supplementary Figure S1. Cell preselection based on optoFGFR receptor level.

Average bitrate in individual cells as a function of mean optoFGFR fluorescence (rolling mean over single-cell trajectories; trajectories in each rolling window have a combined duration equal to 100 times the experiment's duration), superimposed over the histograms of mean optoFGFR fluorescence in individual cells. OptoFGFR levels were log-transformed and standardized within each replicate separately, then pooled by cell line and condition. Cells in the highlighted range were used in further analysis.

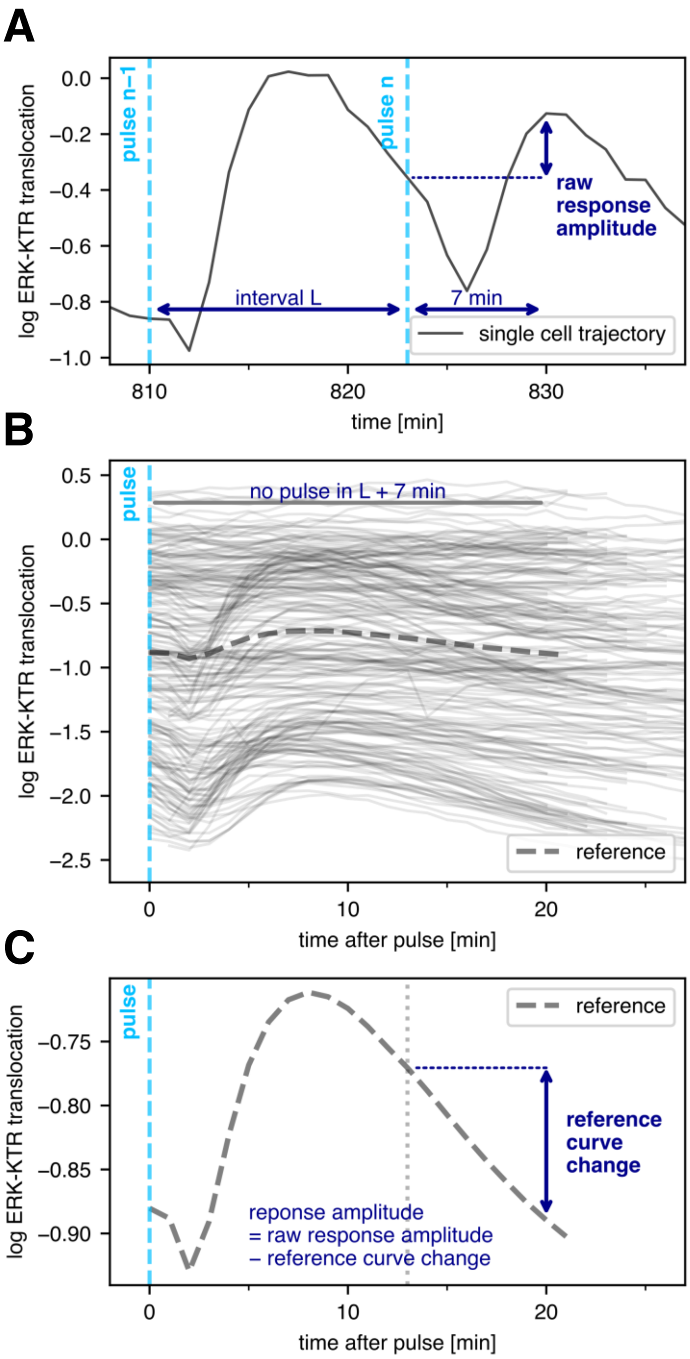

Supplementary Figure S2. Response amplitude definition.

A To compute the response amplitude to light pulse n, which occurred time L after pulse $(n-1)$, we first calculated the raw response amplitude as the difference between the ERK-KTR trajectory at pulse and 7 min after.

B We constructed a reference trajectory by averaging the ERK-KTR trajectories after all pulses not followed by another pulse within the next L + 7 min.

C We computed the change in the reference trajectory between time L and (L + 7 min) after the pulse. The response amplitude was calculated by subtracting the obtained value from the raw response amplitude.

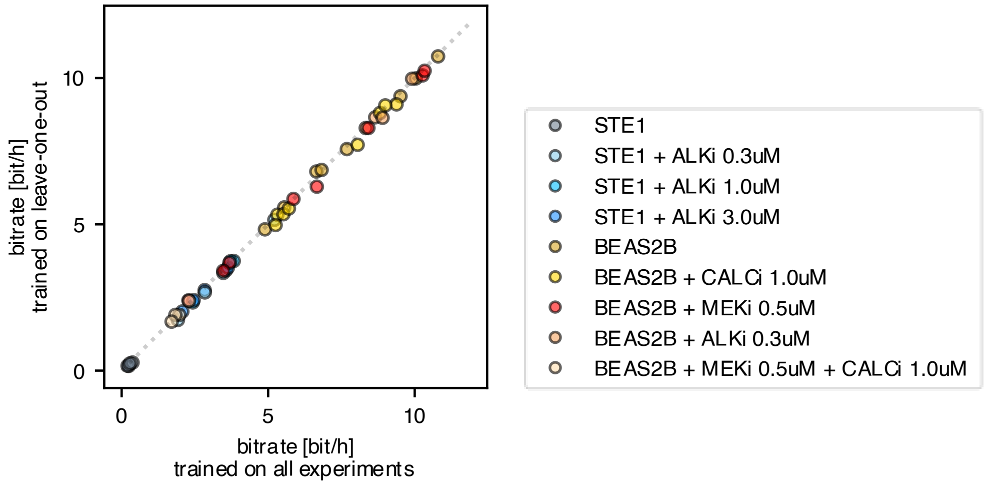

Supplementary Figure S3. Leave-one-out network evaluation.

Average bitrate in particular replicates, computed based on predictions by MLP trained on all conditions and replicates (as used throughout the paper; x-axis), and on all conditions and replicates except for the currently evaluated replicate (y-axis). Identity line dotted.

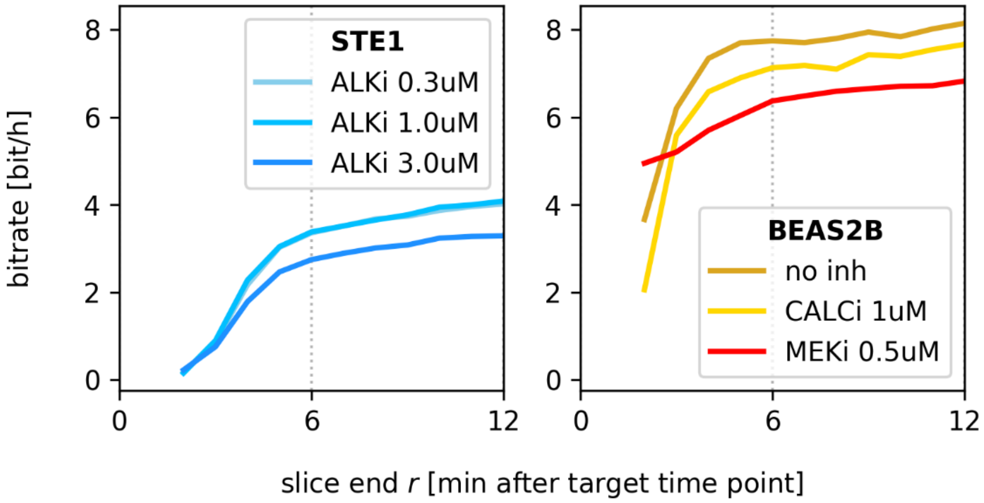

Supplementary Figure S4. Bitrate estimated using MLP trained on slices of increasing length.

The slice used to predict whether there was a pulse at time point *k* in cell *j* is $y^j_{k\ldots k+r}$, i.e., *r* consecutive differences $\Delta y^j_{k+1\ldots k+r}$.

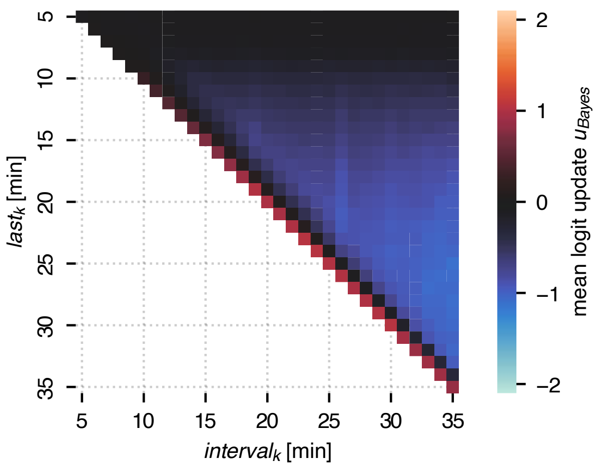

Supplementary Figure S5. Neural network predictions.

Mean logit Bayesian update u_{Bayes} as a function of $last_{\text{k}}$ and $interval_{\text{k}}$ evaluated on STE1 cells with ALKi. The diagonal ($last_{\text{k}} = interval_{\text{k}}$) corresponds to timepoints in which a pulse occurred; the u_{Bayes} values on the diagonal are presented in Fig. 6D.

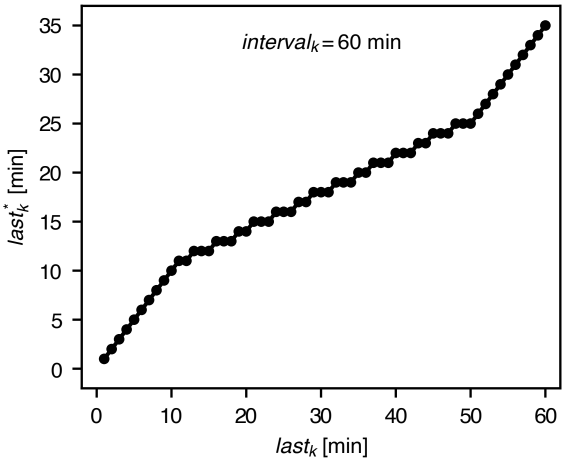

Supplementary Figure S6. Imputation of responses after intervals not present in the dataset.

As intervals longer than 35 min were not present in the experimental sequence, responses for ($interval_k$, $last_k$) with $interval_k$ > 35 min were sampled from data for ($interval_k^*$, $last_k^*$), where $interval_k^*$ = 35 min. The plot illustrates the mapping of $last_k$ to $last_k^*$ in the case of $interval_k$ = 60 min.

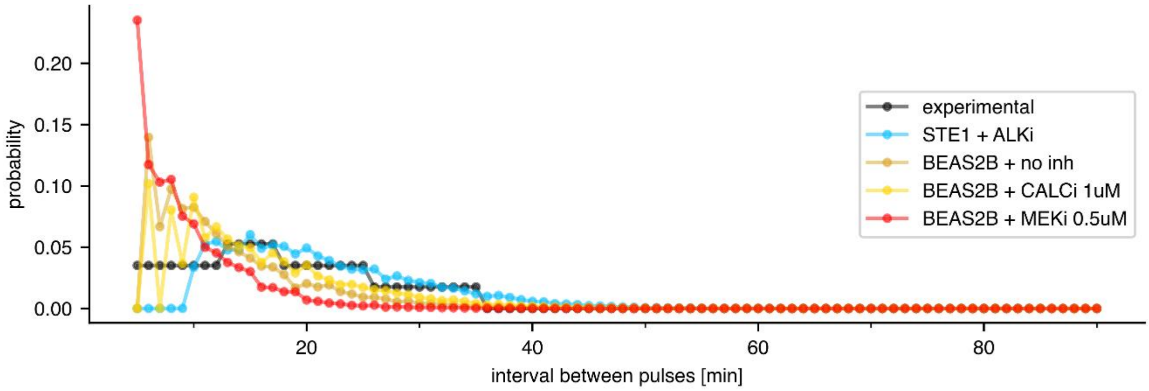

Supplementary Figure S7. Input interval distribution optimized without regularization.

Setup as in Fig. 6E.

Sequence of intervals between light pulses (in minutes):

```
| 35 | 19 | 30 | 34 |  5 | 14 |  5 | 19 | 33 | 14 |  8 |  6 | 18 ↲
| 26 |  6 | 15 | 32 | 15 | 15 | 31 | 17 | 29 | 18 | 17 | 14 |  7 ↲
| 13 |  9 | 10 | 20 | 20 |  9 | 25 | 21 |  7 | 16 | 16 | 27 | 16 ↲
| 28 | 13 | 21 | 25 |  8 | 17 | 13 | 24 | 10 | 11 | 22 | 22 | 23 ↲
| 24 | 12 | 23 | 11 | 12 |
```

Intervals preceding interval of given length:

interval	*preceding intervals*		
**5**	14	34	
**6**	8	26	
**7**	14	21	
**8**	14	25	
**9**	13	20	
**10**	9	24	
**11**	10	23	
**12**	11	24	
**13**	7	17	28
**14**	5	17	33
**15**	6	15	32

interval	*preceding intervals*		
**16**	7	16	27
**17**	8	18	31
**18**	6	29	
**19**	5	35	
**20**	10	20	
**21**	13	25	
**22**	11	22	
**23**	12	22	
**24**	13	23	
**25**	9	21	
**26**	18		

interval	*preceding intervals*		
**27**	16		
**28**	16		
**29**	17		
**30**	19		
**31**	15		
**32**	15		
**33**	19		
**34**	30		
**35**	∞		

Supplementary Table S1. Sequence of light pulses used in experiments.
